# Beyond growth and density: Recentring the demographic drivers of urban health and risk in the global south

**DOI:** 10.1177/00420980211014410

**Published:** 2021-06-09

**Authors:** James Duminy

**Affiliations:** University of Bristol, UK

**Keywords:** COVID-19, family planning, global south, urban demography, urban health

## Abstract

Debates within urban studies concerning the relationship between urbanisation and infectious disease focus on issues of urban population growth, density, migration and connectivity. However, an effective long-term risk and wellbeing agenda, without which the threat of future pandemics cannot be mitigated, must also take account of demographic forces and changes as critical drivers of transmission and mortality risk within and beyond cities. A better understanding of the dynamics of fertility, mortality and changing age structures – key determinants of urban decline/growth in addition to migration – provides the foundation upon which healthier cities and a healthy global urban system can be developed. The study of how basic demographic attributes and trends are distributed in space and how they interact with risks, including those of infectious disease, must be incorporated as a priority into a post-COVID-19 urban public health agenda. This perspective concurs with recent debates in urban studies emphasising the demographic drivers of urban change. Moreover, it raises critical questions about the microbial and environmental emphasis of much research on the interface of urban health and governance.

## 摘要

城市研究中关于城市化和传染病之间关系的争论集中在城市人口增长、密度、迁移和连通性等问题上。然而，一个有效的长期风险和福祉议程（如果没有这样的议程，未来流行病的威胁就无法减轻）还必须考虑人口因素及其变化，因为它们是城市内部以及跨城市传播和死亡风险的关键驱动因素。更好地理解生育率、死亡率和不断变化的年龄结构的动态（除了移民以外，这些是城市衰落/增长的关键决定因素）能为发展更健康的城市和全球城市体系奠定基础。必须将基本人口属性和趋势的空间分布、以及它们与包括传染病在内的各种风险之间的相互作用的研究作为优先事项纳入后新冠肺炎时代的城市公共卫生议程。这一观点与最近城市研究中强调城市变化的人口驱动因素的主张一致。此外，本文还提出了许多关于城市健康与治理方面的、与许多研究的微生物和环境重点相关的关键问题。

## Introduction

As renewed attention is given to an urban health and development agenda for a post-pandemic world, we should place greater emphasis on the demographic drivers of urban risk and resilience in settings of the global south. The task is urgent for at least two reasons. First, natural increase exerts a strong influence on urban growth in the global south. In many cases, natural increase exerts a stronger effect on urban growth than migration, even if the relationship between these factors is highly specific to context, and data limitations make conclusive statements difficult (Fox, 2017; Jedwab et al., 2017; Montgomery et al., 2003). Second, there is now convincing evidence of stalls and reversals in the decline of urban fertility rates in some sub-Saharan African countries (Sánchez-Páez and Schoumaker, 2020).

Recognising the significance of demographic forces and effects will enable us to broaden our post-pandemic urban response to encompass different kinds of interventions – ones not typically engaged in by the urban sector – as mechanisms of alleviating public health risks. Such interventions include reproductive health and family planning programmes. In global south contexts, these tend to fall within the ambit of wider public health programmes, often with a rural focus as many national governments and international donors continue to prioritise rural development through their funding and operations (Shawar and Crane, 2017). The recognition that reproductive health and family planning require specific kinds of responses in urban contexts, and may form an integral part of wider strategies for sustainable urban development, is long overdue.

For many scholars and commentators, the COVID-19 pandemic offers an opportunity to rethink and reshape the ways in which cities everywhere are planned, are managed and function (Lee et al., 2020). We should welcome the chance to recentre a global urban health agenda that, among other things, attends to the ways in which design, mobility and the urban built environment impact health. Yet we should also take heed of calls for a post-pandemic agenda to move beyond disaster response, to encompass the long-term needs of those living in deprived urban conditions of the global south (Corburn et al., 2020). Here I urge caution against the uptake of an urban health agenda framed purely in microbial and environmental terms. Our ideas and responses must include some of the long-term factors that make urban populations more or less vulnerable or resilient to stresses and shocks – health, economic, climatic, demographic or otherwise.

While I will argue that demographic processes significantly impact urban health risk and resilience, public discourse surrounding the COVID-19 pandemic has focused on the role of density, (a simplistic view of) population growth, migration and connectivity as drivers of disease risk and transmission. To date, demographic dynamics and interventions have been notably absent from discussions around anti-pandemic responses (UN-Habitat, 2020), as they are from urban policy debates more generally. Likewise, they have received little attention within urban health, a field that is essentially concerned with the study of the determinants of health and disease in urban areas, including the role of the urban environment in shaping health outcomes (Galea et al., 2019). Given this, one way in which the post-pandemic urban opportunity should be seized is by recognising the significance of demographic forces and changes, their spatial distributions, as well as interventions that help us to realise positive demographic and health outcomes.

## The problematisation ofpost-pandemic urban health

Urban density – understood in terms of both the built environment and population – has emerged as a contentious issue in current discourse on the relationships between urbanisation and infectious disease, but discussions have largely overlooked or taken a simplistic view of its demographic drivers. Density has always occupied an ambivalent status in urban and planning discourse, but the initial months of the global COVID-19 pandemic saw density recast in a negative light that harked back to the public health concerns of the 20th century, when it was regarded as a facilitator of contagion and social pathologies of many forms (Parnell, 1993).

Critical views of urban densification tend to express a degree of physical determinism and an overemphasis on urban form as a driver of health risk. Density is seen as something to be regulated more effectively through appropriate planning and design, land regulation, allocation of public open spaces, widened pavements, the provision of dedicated bus and cycle lanes, and so on. Alternatively, it may be cast within the frame of urban population density. In doing so, urban health is problematised within a Malthusian spatial rubric – an average distribution of population within a bounded space, imposing a limit on population growth through epidemiological risk rather than the direct effects of resource scarcity; an urban corollary of past anxieties and debates surrounding population density that encouraged practical responses in the form of ‘redistribution’ (Bashford, 2007). With epidemiological risk written in the terms of socio-spatial determinism, it is little wonder that some early reactions to the pandemic suggested that cities might have to ‘de-densify’ in a post-pandemic world (Shenker, 2020), where this is understood as the redistribution of population and structural form.

Researchers have tempered those early anxieties. Evidence from the United States indicates that the relationship between density and COVID-19 transmission is mediated by numerous socioeconomic, demographic and infrastructural factors. Indeed, relative to density, connectivity within and between places may have exerted a stronger influence on epidemiological spread and mortality (Carozzi et al., 2020; Hamidi et al., 2020). The role of connectivity may help to explain why many dense urban environments in the global south, including in sub-Saharan Africa, have suffered lower COVID-19 morbidity and mortality than cities in the global north. Moreover, increased density can facilitate access to healthcare and the management of social distancing measures, as demonstrated by East and South-east Asian cities like Singapore and Seoul. Density remains an important urban advantage, although how to promote the ‘right kind’ of density, which maximises socioeconomic productivity while lessening epidemiological risk, will remain a challenge.

Most relevant for this discussion is the fact that pandemic-inspired debates surrounding the health effects and regulation of urban density have tended to overlook some of its specific demographic determinants. Where demographic change does feature, it is usually in the form of a totalising notion of population growth. For instance, scholars point to the increased epidemiological risk arising from population and urban growth, particularly when and where such growth outstrips infrastructure investments and service delivery, thereby promoting the emergence of materially deprived urban peripheries (Coker et al., 2011; Connolly et al., 2020). Such risk is deeply linked to urban inequality and the urbanisation of poverty, which facilitate segregation and leave residents of urban and peri-urban informal settlements particularly vulnerable (Ahmed et al., 2019; Corburn et al., 2020). Yet in these discussions rapid population growth itself is taken as a given; the specific aspects of fertility, mortality and structural demographic change – and the factors influencing those dynamics – are excluded from the epidemiological purview.

A second seam along which commentators have traced epidemiological risks concerns population mobility and connectivity. While domestic migration has been linked to the diffusion of COVID-19 and other diseases, from a broader urban perspective the status of some cities as hubs of global trade and travel has underpinned their role as ‘conduits of transmission’ (Lee et al., 2020). Urban scholars have been well aware of how epidemiological risks are heightened by the realities of global capitalism, with its dependence on migrations of labour and flows of goods and capital to and from global cities (Ali and Keil, 2008). This work is right to highlight the economic, sociopolitical and biophysical context of an increasingly urban and interconnected world, with its implications for public health, the topological nature of disease transmission and the ex-urban infrastructures that are integral to urban function, flows and (ill-)health (Connolly et al., 2020). However, a focus on global cities and supranational patterns of connection and flow arguably underplays some of the most influential urban dynamics of the present. These include fundamental place-based drivers of urban change and wellbeing, such as reproductive health and fertility rates, in cities and neighbourhoods of the global south. These kinds of forces are of particular salience in urban settings that are not so closely bound into global circuits of capital and working bodies as London, Singapore or Tokyo.

There is a danger in maintaining a demographic model focused solely on population growth and migration as an explanation of urban health risk in a post-pandemic world. Total population growth and mobility are necessary but not sufficient factors to explain why some groups or areas may be more or less susceptible to disease outbreaks and wider health problems. Rather, we need a more detailed perspective on how demography relates to urban health – one that includes, for example, the effects and spatial distributions of fertility, mortality and age structure. By taking up this view, reproductive health and family planning services – targeted at spaces within and beyond cities – will emerge as key interventions to address long-term drivers of health risk and to promote sustainable development more generally (Starbird et al., 2016).

In restating the links between demographic and urban change (Montgomery et al., 2003), I will highlight critical pathways through which reproductive health and family planning interventions will promote favourable urban health and development outcomes. I identify five domains (public health, economic, spatial, demographic and environmental) in and through which demographic factors are linked to heightened risks of poor health in cities. These domains are discussed as they pertain to the realities of the global south, where urban and overall population growth are and will be concentrated in decades to come. Particular emphasis is placed on sub-Saharan Africa as the region with the fastest rates of population growth and the highest fertility rates globally (United Nations, 2019).

## Underlying public health risks

Urban areas and groups affected by underlying problems of infectious disease, non-communicable disease and maternal and child ill-health are more vulnerable to epidemiological outbreaks and mortality. While it is true that poor urban populations of the global south usually bear multiple kinds of health risks simultaneously (Ahmed et al., 2019; Stephens, 1996), reproductive health problems present a significant source of urban ill-health and mortality. Young urban women, and particularly those who are poor or living in slums, are at particular risk of falling pregnant unintentionally (Faye et al., 2013; Ikamari et al., 2013). Many resort to abortion services that may be unsafe and result in complications. In Malawi, for example, a significant proportion of poor pregnant women in urban areas procure abortions from traditional healers (39%) or perform it themselves (14%); over half of all procedures result in health complications, with a little under a fifth of those going untreated (Polis et al., 2017). We know that poor urban communities often face considerable barriers to their use of maternal care services (Rossier et al., 2014). In some cases, access to these services may be worse for slum residents than for the rural poor (Matthews et al., 2010). In a context like Kenya, this results in considerable excess maternal mortality in urban slums relative to the national average (Rossier et al., 2014).

Despite the significance of reproductive health problems in urban settings of the global south, they are almost entirely absent from urban policy discussions (UCLG, 2019; United Nations, 2017b). Given this silence, it is unsurprising that interventions like voluntary family planning programmes are also excluded from considerations of urban development. Yet increasing access to family planning services has demonstrated the potential to promote better health outcomes, in particular by reducing mortality among mothers and children through preventing unintended and high-risk pregnancies. It is estimated that eliminating unmet need for contraception – the proportion of women who want to avoid or delay another birth but who are not using any method of contraception – would reduce maternal deaths in developing countries by 30% (Cleland et al., 2012).

Moreover, by increasing the time interval between pregnancies, increased use of family planning has the potential to improve perinatal outcomes and child survival. In developing countries, if all childbirths were separated by a gap of two years the risk of death in infancy (less than one year of age) would fall by 10%; that risk would decrease by 21% for those between one and four years old (Cleland et al., 2012). Effective family planning programmes will also contribute to better women and child health outcomes through improved nutrition (Naik and Smith, 2015). Indeed, increasing access to family planning has been identified as a key strategy to address the underlying causes of child undernutrition in urban informal settlements (Goudet et al., 2017) and to close urban–rural gaps in child malnutrition (Ervin and Bubak, 2019). These issues take on particular salience in the urban context, as urban consumers are disproportionately affected by food price shocks and disruptions to supply chains like those introduced by the COVID-19 pandemic.

## Economic development and poverty reduction

Societies and urban populations affected by higher levels of poverty and lower wellbeing, in which women are less empowered, and where there are greater income gaps between the rich and poor, are likely to face greater health risks from epidemics and other sources (Sohler et al., 2003; Stephens, 1996; Wilkinson and Pickett, 2009). That poverty, income inequality, education, as well as working and housing conditions are significant drivers of poor health outcomes in cities and slum areas is a key insight of the literature addressing the social determinants of health (Harpham, 2009; Lilford et al., 2017; Weimann and Oni, 2019). Gender inequality is of particular importance given that epidemics may have particular kinds of impacts on men and women (UNDP, 2020; Wilkinson et al., 2020). Some arise from women’s prominent role in urban and cross-border informal food trades (Brenton et al., 2013). Others are felt through inhibited access to sexual and reproductive health services (Riley et al., 2020).

Family planning programmes can promote positive outcomes for urban poverty reduction and economic growth in at least three ways. First, by lowering fertility rates, family planning can help to boost the proportion of the population that is of working age, decreasing the dependency ratio and enabling greater numbers of women to participate in paid labour (Bongaarts and Sinding, 2011; Canning and Schultz, 2012). Indeed, economists have recognised the importance of well-designed and context-relevant family planning programmes as part of wider efforts to reduce fertility and promote economic growth in sub-Saharan Africa through the ‘demographic dividend’ (Bloom et al., 2013, 2017). While scholars have critiqued the assumptions and realisability of the dividend hypothesis (Cleland and Machiyama, 2017; Lutz et al., 2019), it is nonetheless the case that a reduction in the youth dependency ratio facilitated by family planning investments would have positive effects if pursued alongside a range of other socioeconomic investments.

Second, family planning can help to reduce the burden imposed on urban communities and community-level facilities by rapidly growing populations. One aspect of this benefit includes significant economic savings in public expenditure. It is estimated that each dollar invested in family planning saves over two dollars in pregnancy-related care, and even more when other development sectors are considered (Bongaarts and Sinding, 2011; Guttmacher Institute and BMGF, 2017). These savings could be reinvested in other health-promoting urban infrastructures and programmes.

Third, at the individual and household level, controlled trials have demonstrated that increasing access to family planning services can substantially improve women’s earnings and assets by avoiding the additional expenses associated with having unintended children (Canning and Schultz, 2012). Family planning investments not only have powerful poverty-reducing effects but also can help to reduce economic inequalities between women, as poorer and less-educated urban women experience greater rates of unintended pregnancy and face more significant barriers in accessing contraception than their better-educated and wealthier counterparts (Bongaarts and Sinding, 2011; Faye et al., 2013; Ikamari et al., 2013).

## The spatial form of cities and urban poverty

Slum settlements are perhaps the most obvious spatial manifestation of urban poverty and inequality in the global south, even if they are not the sole loci of urban deprivation (Montgomery, 2009). Slum residents face significant health risks in general (Ezeh et al., 2017). Their vulnerabilities to outbreaks of contagious diseases like COVID-19 are heightened by a lack of access to clean water and healthcare facilities, the propensity for overcrowding in small structures, high levels of social mixing, and the fact that their populations are often highly mobile or transient (Corburn et al., 2020).

Slum populations would stand to benefit most from the health-improving and economic effects of family planning, but their access to services is often constrained. Slum residents in many sub-Saharan African countries, for example, have a far greater unmet need for contraception than those living in wealthier urban areas, and sometimes this need is even greater than that of rural populations (Ezeh et al., 2010; Fotso et al., 2013). In Kenya, for example, while the Nairobi region enjoys the lowest levels of unmet need nationally, women residing in slum areas of the capital city have a need that is double that of the city as a whole and higher than that of rural women (Beguy et al., 2017). While a greater proportion of poor urban women might use contraception relative to rural women, unmet need can still be higher for the former group. Reasons for this include greater demand to limit childbearing in poor urban areas, where costs of household and social reproduction are relatively high and where rhythms of life and income may be particularly unpredictable (Machiyama et al., 2019). Moreover, poor urban women experience significant geographic and economic barriers to family planning access – the supply of services does not necessarily penetrate into poorer neighbourhoods, leaving local residents often dependent on private providers issuing services of variable quality and relatively high cost (Ezeh et al., 2010).

As discussed above, increasing access to family planning services would introduce significant health and economic benefits for urban women, children, households and communities. Moreover, there is evidence to suggest that effective family planning programmes implemented by poor countries at the national level, by lowering rates of population growth, would significantly slow increases in the national urbanisation rate, reduce the share of the total urban population living in informal settlements, decrease overall urban density and boost total welfare levels (Jedwab and Vollrath, 2019). This does not amount to an endorsement of family planning as a means to prevent rural–urban migration and urban growth. Rather, it should be recognised that urban-focused family planning interventions, when pursued alongside wider programmes that also benefit rural populations, will result in generally positive outcomes for cities and communities.

## The demographic structure of cities

Demographically, urban settings with more or less aged or youthful populations are likely to experience health risks in various ways and to differing extents. It is clear, for example, that the risk of dying from COVID-19 increases with age, with most deaths occurring among people aged over 60 and particularly those with pre-existing chronic health conditions. This is of particular concern for countries of the global south, given that 69% of the global population aged 60 or above reside in low- and middle-income countries (Lloyd-Sherlock et al., 2020). What is important here is not only the demographic structure of age cohorts, but also their spatial distributions. In China, for instance, there is evidence to suggest that the increased density of the older population was linked to high rates of COVID-19 morbidity in urban areas (You et al., 2020).

While there is a need to consider how demographic structure affects health and wellbeing in cities, this is currently not well understood for the global south. Much of the development and health research and policy focus on Africa, for example, is concerned with its youthful population. For the continent’s cities, of particular interest is the existence of a ‘youth bulge’ resulting from low urban fertility rates (relative to rural areas) combined with youthful migration into towns and cities (Sommers, 2010; United Nations, 2017a). Some have hypothesised that youthfulness is one reason why African populations have ostensibly enjoyed lower COVID-19 morbidity and mortality than other regions with higher median ages (Njenga et al., 2020). That said, in African contexts, older populations are also significant and growing in proportion (Pillay and Maharaj, 2013). While the issue of ageing in cities has been taken up in the urban and planning literatures in the global north, the implications of both high fertility rates *and* ageing in African cities, often occurring in conditions of urban poverty, are notably absent from the literature (Aboderin et al., 2017). Family planning could play a key role in ensuring appropriate support ratios within urban societies, but this remains to be explored in any detail.

## Environmental risk and resilience

Urban areas and groups that are less equipped to mitigate or adapt to climatic or environmental stresses and shocks will experience heightened vulnerability to public health threats and emergencies. Here too, family planning can potentially play a beneficial role. Proponents of voluntary family planning programmes argue that slowing global population growth by meeting unmet need for contraception could have substantial climatic co-benefits by reducing emissions (Bongaarts and O’Neill, 2018). At the same time, critics see a family planning agenda as detracting attention from the harm caused by consumption-intensive development and as having limited potential to impact global climate change (Martine, 2009). Regardless of these debates, it is true that fertility rates exert a powerful influence on urban growth. Increasing access to family planning is therefore critical for easing the adaptation burden both generally and in the urban context (Bongaarts and O’Neill, 2018; Bryant et al., 2009). A key question, then, is how family planning could be effectively integrated with wider adaptation and resilience strategies that address the needs and vulnerabilities of the urban poor.

## Conclusion

The COVID-19 pandemic has helpfully raised the urgency and profile of a global urban health agenda, demonstrating that urban change, particularly in the global south, is and will increasingly be central to *global* health risks and responses. However, there is a danger that in articulating a post-pandemic development and health agenda focused on settings of the global south, the significant role of demographic forces and interventions will be overlooked. To be clear, this is not a call for an uncritical return to practices invoking colonial tropes and logics, or to coercive demographic projects like those implemented in some Asian contexts in the later decades of the 20th century. Those critiques, while important, have run their course. Rather, this is an appeal for a long overdue appreciation of the importance of demographic factors in shaping urban areas and wellbeing, and of how rights-based interventions related to reproductive health and family planning (alongside other essential socioeconomic investments) can play a critical role in promoting healthier and more sustainable urban futures.

This argument has relevance for urban scholars and our conceptual understanding of how urban processes are linked to health outcomes. First, in staking out an empirical research agenda, adopting a more detailed perspective on how demography relates to urban health, including risks of public health emergencies, implies further examination of the distributions and interrelated effects of fertility rates, mortality, age structure and healthcare. This perspective accords with a view of urbanisation that emphasises demographic rather than economic processes in accounting for urban trends in the global south (Fox, 2017; Hommann and Lall, 2019; Jedwab et al., 2017). In the same vein, our understanding of the drivers of health risk should not be limited to the economic drivers and consequences of globalisation and global city development. We must attend not only to airports and bus stations, but also to the urban governance of reproductive health services; to local journeys to and from healthcare facilities, as well as international air travel; to the material realities of places and neighbourhoods as much as the global space of flows.

Second, a demographic view invites critical reflection on the ways in which urban health and governance have been and are problematised within urban studies, often in favour of the microbial and environmental domains. Urban scholars have documented the legacies and effects of the virological and ‘bacteriological city’ on contemporary ideas and practices of governance (Gandy, 2006; Keil and Ali, 2007). However, they have devoted less attention to the processes, objects and knowledge surrounding human fertility and reproduction, both past and present, and how these bear upon urban risks, ideas and responses. In thinking through post-pandemic urban futures, it is imperative that we recognise and address such factors in their multiple connections to urban health, socio-cultural life and development. Failing to do so would help to perpetuate a historical disjuncture between the urban and demographic/health sectors, one which we can ill afford given the scale of the urban challenge in a post-pandemic era.
